# Dynamic prediction of cardiovascular risk from linked primary care records in New South Wales, Australia: protocol for a retrospective prognostic modelling study

**DOI:** 10.1136/bmjopen-2026-122980

**Published:** 2026-07-27

**Authors:** Nicholas I-Hsien Kuo, Heidi Welberry, Sanja Lujic, Anna Campain, Daniel Wilson, Katie Harris, Michael Kidd, Patricia Correll, David Peiris, Louisa Jorm

**Affiliations:** 1 Centre for Big Data Research in Health, University of New South Wales, Sydney, Australia; 2 The George Institute for Global Health, University of New South Wales, Sydney, Australia; 3 NSW Ministry of Health, Sydney, Australia; 4 International Centre for Future Health Systems, University of New South Wales, Sydney, Australia; 5 Nuffield Department of Primary Care Health Sciences, University of Oxford, Oxford, UK

**Keywords:** Cardiovascular Disease, Risk Assessment, Primary Health Care, Electronic Health Records, Machine Learning

## Abstract

**Abstract:**

**Introduction:**

Cardiovascular disease (CVD) remains a leading cause of preventable morbidity and mortality. Most existing CVD prediction tools use a baseline survival modelling framework, in which each predictor is represented by a single value recorded at the start of follow-up. Although risk calculators can be re-run using updated values, these models do not explicitly use the longitudinal, irregularly recorded information in primary-care electronic medical records (EMRs). Dynamic modelling approaches have the potential to generate updated CVD risk estimates at each general practitioner (GP) encounter, supporting both risk assessment and monitoring of changes over time. This protocol outlines the development and validation of transformer-based sequence models for encounter-level prediction of 5-year CVD risk, compared with dynamic landmark survival models and benchmarked against static CVD prediction tools.

**Methods and analysis:**

The study will use the New South Wales (NSW) Lumos linked health data asset, a large, privacy-preserving linkage of GP EMRs with hospital admissions, mortality and other administrative datasets across NSW, Australia. Eligible GP encounters will be included from 2018 onwards. The primary outcome is first fatal or non-fatal CVD event within 5 years of each eligible encounter, identified using ICD-10-AM codes in linked hospital and mortality data. Predictor variables include routinely collected demographics, chronic conditions, clinical measurements and medications, as well as healthcare utilisation patterns available at or before each encounter. Sex-specific dynamic landmark survival models (such as Cox proportional hazards models) with time-updated covariates (full and least absolute shrinkage and selection operator (LASSO)-regularised) will be developed alongside sex-specific transformer-based sequence models, fine-tuned for 5-year risk prediction at each encounter. Internal validation will use repeated resampling for landmark survival models with patient-level and temporal separation for sequence models. Geographic transportability will be evaluated using internal–external validation across primary health networks (PHNs). Performance will be evaluated using discrimination, calibration, decision-curve analysis and subgroup analyses and will be compared with existing static predictive models. Missing-data will be imputed where necessary: a range of imputation approaches will be compared to optimise computational efficiency but also support predictive generalisability.

**Ethics and dissemination:**

This study is conducted under NSW Health governance arrangements with approval from the NSW Population and Health Services Research Ethics Committee (2019/ETH00660). Analyses will be performed within a secure data environment using de-identified linked data. Results will be reported in accordance with TRIPOD-AI guidance and disseminated through peer-reviewed publications, scientific conferences and policy and consumer forums.

**STRENGTHS AND LIMITATIONS OF THIS STUDY**

- This study uses the New South Wales Lumos data asset, a large, statewide privacy-preserving linkage of general practice electronic medical records with hospital, mortality and other health datasets, enabling population-scale modelling of cardiovascular risk trajectories across metropolitan, regional and rural settings.
- The protocol prespecifies and compares two complementary dynamic modelling approaches aligned to the same encounter-level prediction objective, using the same underlying patient historical records: dynamic landmark survival models and transformer-based sequence modelling.
- The study incorporates routinely recorded quantitative clinical measurements, including pathology results, body mass index and blood pressure, allowing more granular modelling of risk trajectories than approaches based primarily on coded events alone.
- Validation is designed to address real-world implementation challenges through patient-level and temporal separation, internal–external validation across primary health networks (PHNs), decision curve analysis and prespecified subgroup and equity analyses.
- Predictor availability and data completeness may vary across practices and PHNs; although PHN-specific inclusion dates and alternative missing-data strategies are planned, residual variation in recording quality may still affect model performance and transportability.

## Introduction

 Cardiovascular disease (CVD) remains the leading cause of potentially preventable morbidity and mortality in Australia, accounting for substantial clinical burden and health-system expenditure across all stages of care.^1 2^ For the purposes of risk prediction, CVD refers to a composite of major cardiac, cerebrovascular and peripheral vascular conditions, including myocardial infarction, stroke, peripheral arterial disease and heart failure. Early identification of individuals at elevated CVD risk is central to effective prevention, enabling timely initiation of lifestyle, pharmacological and system-level interventions.^3–5^ Risk prediction tools are therefore a cornerstone of primary prevention strategies in contemporary primary care.

Despite their widespread use, most established CVD risk prediction models are developed using static baseline survival modelling frameworks. In these frameworks, each individual typically contributes a single value for each predictor, recorded at or near the start of follow-up, and subsequent changes in risk factors during follow-up are not explicitly modelled. In clinical practice, tools such as PREDICT (New Zealand),^6^ QR4 (UK),^7^ PREVENT (USA)^8^ and AusCVDRisk (Australia)^9^ can be re-run using the most recently available observations. However, the underlying models generally do not incorporate the longitudinal history of prior measurements, diagnoses, medication changes, consultation timing or gaps in recording. These features are central to primary-care electronic medical records (EMRs), where diagnoses accumulate, medications are initiated and discontinued, laboratory tests are ordered selectively, and consultation patterns themselves may reflect emerging illness.^10–14^ Dynamic modelling approaches assess whether using information accumulated up to each encounter improves prediction beyond simply updating a conventional model with the latest available values. Although dynamic and machine learning–based prediction models have been proposed,^15–17^ there remains limited evidence on their performance, transportability and clinical utility in real-world primary care settings.^18^

The New South Wales (NSW) Lumos data asset^19^ provides an unprecedented opportunity to address these limitations at population scale. Lumos is a statewide, privacy-preserving linkage of routinely collected general-practice electronic health records with hospital admissions, emergency department presentations, mental health services, ambulance records and mortality data across NSW, Australia. It includes the health records of over five million individuals since 2010—representing nearly half the state’s population over this period—drawn from more than 800 general practices.^20^ The population aged 18–84 years and CVD-free is estimated to be over 4.1 million per calendar year since 2018. The breadth, longitudinal depth and linkage across care settings make Lumos particularly well suited for developing and validating real-world cardiovascular risk prediction models.

Recent work using Lumos has demonstrated that sex-specific Cox proportional hazards models using routinely collected primary care EMRs can achieve good discrimination and calibration for 5-year CVD risk prediction.^21^ However, these models used a single index date and were not designed to estimate how risk changes across successive general practitioner (GP) encounters. Dynamic survival modelling approaches, including landmark modelling, have been proposed to address this limitation by updating risk predictions at clinically relevant time points using information available up to each landmark.^15^ Landmark models estimate the risk of an event within a fixed prediction horizon conditional on being event-free at the landmark time, enabling alignment between prediction and clinical decision points.^22^ In this study, landmark models will provide a transparent way to compare the benefit of using time-updated covariates with the benefit of more complex sequence-based modelling. By generating updated risk estimates at each consultation, these approaches also enable quantification of changes in predicted risk following lifestyle modification or optimisation of risk factors (eg, blood pressure, lipids or glycaemic control). This supports ongoing monitoring and potentially reinforces patient engagement with preventive strategies, as well as facilitating more appropriate ‘what-if’ scenario analyses using updated risk factor profiles, reflecting common clinical use of risk calculators to explore the impact of risk factor changes.

Internationally, methodological advances have increasingly shifted attention toward dynamic, sequence-based approaches that treat EMRs as ordered longitudinal data. Transformer architectures,^23 24^ including clinically adapted models such as Bidirectional Encoder Representations from Transformers for Electronic Health Records (BEHRT),^25^ have shown strong capacity to learn from irregular clinical histories and capture long-range temporal dependencies. Recent work in the UK extended BEHRT to a survival modelling framework (TRisk) using primary care electronic health records from the Clinical Practice Research Datalink, demonstrating the feasibility of transformer-based CVD risk prediction at population scale.^26^ In that study, risk was estimated from a single randomly selected baseline time point within each patient’s history and model inputs were primarily derived from coded events, including diagnoses, medication categories, laboratory test ordering (rather than measured results) and procedure codes.

This protocol outlines a prespecified, TRIPOD-AI-aligned^27^ framework to develop and validate a dynamic, sequence-based CVD risk prediction model using the NSW Lumos data asset. The proposed approach models primary-care EMRs as longitudinal encounter sequences to generate updated cardiovascular risk predictions at each GP encounter, reflecting the evolving clinical information available in routine care. This study will incorporate sociodemographic information, diagnoses, lifestyle behaviours but also multiple individual quantitative clinical measurements over time—such as laboratory test results, body mass index and blood pressure values—captured within EMRs, enabling highly granular representation of patient risk trajectories. Model performance will be compared with dynamic landmark survival models, which provide a regression-based comparator aligned with the same encounter-level prediction task and with existing risk tools or previously developed Lumos static models applied using current encounter-level information where feasible. Leveraging the scale and cross-sector linkage of Lumos, the study will also evaluate model performance across geographically distinct primary-care regions to assess transportability in real-world health-system settings.

The aim of this study is to develop and validate an encounter-level, transformer-based sequence model for predicting 5-year cardiovascular risk using linked EMR data from the NSW Lumos data asset and to compare its performance against dynamic landmark survival models and benchmark approaches that use the latest available values at each encounter. By separating, where possible, the effects of time-updated predictors from the additional effects of longitudinal trajectory modelling, the study aims to quantify the marginal value of dynamic sequence modelling for clinically relevant CVD risk prediction in primary care.

## Methods and analysis

### Study design and setting

This is a protocol for a retrospective prognostic modelling study using routinely collected, linked electronic health record data from the NSW Lumos data asset.

Lumos includes linkage of GP EMRs from over 800 GP practices in NSW, Australia, with hospital admissions, emergency department presentations, mental health ambulatory care, ambulance data and mortality records.^19^ These linkages enable longitudinal follow-up across care settings and support ascertainment of predictors, outcomes and censoring. Record linkage is performed by the Centre for Health Record Linkage^28^ using privacy-preserving probabilistic methods. All analyses will be undertaken within a secure analytics environment using de-identified data.

### Study status and timeline

At the time of submission (June 2026), the study is in the data preparation and analysis planning stage, with data access and secure environment arrangements in place. Analyses are expected to be completed by end 2028.

### Patient and public involvement

Patients and members of the public were not directly involved in the design of the modelling methods or selection of statistical approaches for this study. However, consumer perspectives have informed the broader Lumos programme and this research project through representation on project governance and advisory structures, including a Steering Committee with consumer representation, as well as GP and GP software expertise, and a dedicated Consumer Reference Group. These structures contribute to prioritisation of research questions, oversight of data use, interpretation and communication of findings and consideration of community expectations regarding privacy, data sharing and public benefit.

The focus on developing practically implementable, encounter-level risk prediction tools reflects the need to support patient–clinician discussions in routine care, including communication of cardiovascular risk and monitoring of changes over time. Findings from this study will be disseminated in formats accessible to both clinical and community audiences, including plain-language summaries where appropriate.

### Study population and sample selection

The unit of analysis will be the individual GP encounter. The study is designed to support dynamic risk estimation at the point of care; accordingly, each eligible GP encounter will serve as a prediction landmark. Prediction performance will be considered at both the encounter and person level as discussed further in the model performance assessment section.

Encounters will be eligible for inclusion if they occur in a Lumos practice and meet all of the following criteria at the time of encounter: the patient is aged 18–84 years, has no prior history of CVD and has at least one current or prior anthropometric or pathology measurement recorded to support risk estimation. Encounters for individuals with unresolvable conflicting values across datasets for key variables such as age, sex or date of death will be excluded as these likely reflect data or linkage errors.

To allow an adequate timeframe for each patient trajectory prior to prediction, encounters will be included only from 2018 onwards. Data extraction methods differ across participating Primary Health Networks (PHNs); therefore, eligible encounters will be included only from a PHN-specific start date, dependent on the available data extracted within each PHN. This approach is intended to reduce bias arising from incomplete early capture of clinically important predictors.

### Predictors to be measured

Candidate predictors will be selected a priori based on prior CVD risk prediction literature,^6–9 21^ clinical relevance and routine availability in Lumos general practice data. Only information recorded at or before each prediction landmark will be used. Sex-specific models will be developed based on a consistent set of candidate predictors.

Candidate predictors will include demographic variables, lifestyle factors, chronic conditions, medication exposures, clinical measurements and healthcare utilisation patterns. Demographic variables will include age, area-level socioeconomic disadvantage using Index of Relative Socioeconomic Disadvantage quintiles,^29^ and PHN. Indigenous status, ethnicity, languages spoken and individual-level socioeconomic status are not available in Lumos data; limiting equity assessment. Lifestyle factors will include smoking status, acknowledging that routine EMR smoking data may be incomplete or out of date; other factors such as alcohol consumption are not reliably recorded in Lumos data. Chronic conditions will include major cardiometabolic, respiratory, mental health conditions derived from coded disease flags. Medication exposures will include total number of medications as well as specific flags for antihypertensives, lipid-lowering therapies, anticoagulants, glucagon-like peptide-1 receptor agonists and other relevant drug classes identified using Anatomical Therapeutic Chemical coding.^30^ Clinical measurements will include routinely recorded quantitative variables such as glycated haemoglobin, body mass index, estimated glomerular filtration rate, systolic blood pressure, triglycerides, cholesterol ratio, alanine aminotransferase and haemoglobin. Healthcare utilisation measures will include GP visit frequency and regularity, inter-visit gaps, and selected Medicare Benefits Schedule^31^ items such as chronic disease and mental health management plans. Inclusion of prior Hospital or Emergency Department visit patterns will not be incorporated into primary analyses as these data are currently available through linkage only, rather than at the point of GP consultation. Secondary analyses will investigate whether their addition improves prediction.

### Outcome measures

The primary outcome will be first incident fatal or non-fatal CVD event within 5 years of each eligible GP encounter. Outcomes will be ascertained using linked hospital admissions and mortality records within Lumos. Incident CVD will be defined using prespecified International Classification of Diseases, 10th Revision, Australian Modification (ICD-10 and ICD-10-AM) code lists,^32^ consistent with previous Lumos-based CVD studies.^21^ A qualifying event will be identified if a relevant ICD-10 code is recorded as a diagnosis in a hospital episode or as the underlying cause of death.

The primary composite outcome will include major cardiac, cerebrovascular and peripheral vascular events, principally ischaemic heart disease, stroke, heart failure and peripheral arterial disease.^21^ Sensitivity analyses will assess alternative outcome definitions, including narrower and broader composites and inclusion of GP-assigned CVD flags.

### Sample size considerations

The study will use all eligible encounters available within Lumos during the study period. Our prior Lumos analyses^21^ followed 800 000 individuals from a single point in time and observed over 30 000 CVD events. Given the current Lumos programme scale,^19 20^ the available dataset is expected to include an eligible population of more than 4.1 million. We therefore expect substantially more outcome events than our prior study, clearly exceeding minimum recommended thresholds for prognostic survival modelling.^33^ This is expected to provide sufficient precision for model development, internal validation, subgroup analyses and internal–external validation across PHNs.

For the sequence-based models, adequacy of sample size will additionally be assessed empirically through model learning behaviour and stability during training. The use of the full statewide dataset is justified not only on statistical grounds, but also because the study aims to develop models that are clinically relevant, equitable, transportable across regions and sub-groups, and informative for health-system implementation.

### Missing data and preprocessing

Missing data are expected because Lumos reflects routine clinical practice, where measurements are selectively recorded and encounter timing is irregular, and patients may receive care at practices that do not contribute data to Lumos. Missingness will not be assumed to represent absence of clinical activity. For landmark survival analyses, predictors will be defined at the patient-encounter level. Categorical variables will be harmonised to standard categories; absence of a disease flag will be treated as absence of the condition; and missing socioeconomic values will be replaced using the modal quintile within each PHN. Remaining continuous predictors will be handled using either multiple imputation by chained equations or non-imputation approaches based on missingness indicators and clinically meaningful default values. These alternative strategies will be compared to assess the impact of informative missingness on model performance, and to balance statistical rigour with approaches that are computationally efficient and feasible for implementation in clinical systems.

Sensitivity analyses will examine the robustness of these strategies across calendar time and PHNs to assess potential impacts of changes in clinical practice, data capture and population characteristics. Continuity of observation will be described using indicators such as time since first and last contributing-practice encounter, encounter frequency, long gaps between encounters, practice participation period and availability of repeated pathology or anthropometric measurements. Where possible, these indicators will be used to help distinguish likely clinical inactivity within contributing practices from incomplete capture due to care received elsewhere.

For transformer models, no baseline completeness requirement will be imposed beyond study eligibility. Missing continuous values will not be imputed. Instead, unobserved values will be explicitly represented within the model inputs, while irregular timing and selective measurement will be retained as features of the longitudinal data structure or encoded through derived temporal indicators.^34^ This enables the model to learn jointly from observed values, patterns of missingness and intervals between recorded encounters, while recognising that these intervals may reflect a mixture of true clinical inactivity, care outside contributing practices and incomplete data capture.

### Data representations

Two data representations of the same at-risk population will be constructed. The first will structure data at the patient-encounter level for dynamic landmark survival modelling. The second will encode each patient’s history as an ordered sequence of time-stamped clinical events for transformer-based modelling.

### Descriptive analyses

Variables will be summarised at the patient and encounter level using appropriate summary statistics (means (SD), medians (IQR) and counts (%)). Comparisons between encounters with and without subsequent incident CVD (within the prediction horizon) will be presented descriptively; formal hypothesis testing will not be emphasised.

### Model development

For the landmark survival modelling approach, predictors will be updated at each eligible GP encounter and models will estimate 5-year CVD risk conditional on being event-free at that time. Continuous predictors will be transformed where appropriate. Sex-specific models will be fitted to accommodate known differences in CVD risk profiles.

A range of survival modelling approaches will be explored, including Cox proportional hazards models (with and without penalisation using least absolute shrinkage and selection operator (LASSO) regularisation) and alternative flexible time-to-event models. Alternative time scales, including age and time since landmark, will be explored, and competing risks methods will be examined with non-CVD death treated as a competing event.

Comparative analyses will assess the impact of model choice on discrimination, calibration and clinical utility, to identify approaches that balance predictive performance, interpretability and feasibility for implementation. To help separate the sources of any improvement, we will compare: existing or previously developed static models applied using the most current information available at the encounter; landmark models using time-updated covariates; landmark models including summaries of recent change or trajectory where feasible; and transformer-based sequence models using the ordered history. These comparisons will examine whether performance gains arise mainly from updated observations, repeated quantitative clinical measurements, or modelling of trajectories and encounter patterns.

For the sequence-based approach, sex-specific transformer-based models will be developed. Longitudinal histories will be represented as ordered clinical events, including diagnoses, medication changes, pathology tests and clinical measurements. After supervised fine-tuning, the models will be subject to task-based post-training for 5-year encounter-level risk prediction using patient longitudinal records.

### Internal validation

Internal validation will be conducted separately for the two modelling approaches. For landmark survival models, repeated five-fold cross-validation and bootstrap resampling will be used to assess model stability. All preprocessing steps, including imputation and transformation, will be repeated within each resampling iteration.

For sequence-based models, repeated cross-validation is not computationally feasible. Instead, internal validation will use patient-level separation into mutually exclusive training, validation and test sets, with stratification by outcome and PHN. Temporal separation will be enforced within the training process using forward-chaining splits, so that tuning and early stopping are evaluated only on future encounters relative to the training window. This design is intended to reduce information leakage and better approximate real-world deployment.

### Model performance assessment

Model performance will be assessed in terms of discrimination, calibration and clinical utility. Discrimination will be evaluated using Harrell’s C-index and time-dependent area under the curve. Calibration will be assessed using calibration-in-the-large, calibration slope and graphical comparison of observed and predicted risks. Clinical utility will be evaluated using decision curve analysis across clinically relevant risk thresholds, comparing each modelling approach with alternative decision strategies such as treat-all and treat-none.

Given that multiple encounters from the same individual may contribute, performance estimates will primarily be calculated at the encounter level, with sensitivity analyses to assess the impact of within-person clustering, including approaches based on selecting a single encounter per individual or applying weighting schemes. Existing risk algorithms and previously developed Lumos static risk models^21^ will be used as benchmark comparators for all metrics, applying the latest available values at each encounter where feasible to reflect their intended use in practice.

### Internal–external validation and subgroup analyses

Geographic generalisability will be assessed using internal–external validation based on leave-one-PHN-out resampling. In each cycle, one PHN will be held out as a pseudo-external region and models will be derived using the remaining PHNs. For sequence-based models, this will be implemented at the testing stage using the reserved data because repeated refitting across all PHNs is computationally intensive. Limited model updating, such as recalibration or parameter-efficient fine-tuning, will be explored and transparently reported. We will interpret internal–external validation as an assessment of transportability within Lumos rather than as a substitute for fully external validation in an independent data asset.

Because primary models will be sex-specific, subgroup analyses will be conducted within sex strata. Prespecified subgroups will include age group, socioeconomic disadvantage as defined by area-level index of relative socioeconomic disadvantage based on residential address, geographic PHN region and key comorbidity groups. Performance within subgroups will be assessed using the same discrimination, calibration and decision-curve metrics as in the main analyses. Where important disparities are identified, additional analyses such as subgroup-specific recalibration will be explored. We will explicitly acknowledge that the absence of Aboriginal and Torres Strait Islander status, ethnicity, language and individual-level socioeconomic measures limits assessment of equity and may obscure model misspecification in important population groups.

### Implementation considerations

The primary output of this study will be validated risk prediction models and evidence on their comparative performance, calibration, clinical utility and transportability. To assess potential usability in general practice, we will document which predictors are available in the GP record at the prediction encounter, the computational requirements for producing encounter-level predictions, and the extent to which model outputs can be explained to clinicians and patients. We will also compare the dynamic models with simpler approaches so that any incremental performance can be weighed against interpretability, workflow burden and implementation complexity.

### Software

Analyses will be conducted in Python,^35^ with PyTorch^36^ used for custom machine learning model development.

## Ethics and dissemination

Use of the NSW Lumos data asset for this study is conducted under NSW Health governance arrangements and is covered by existing program-level approval from the NSW Population and Health Services Research Ethics Committee (approval ID: 2019/ETH00660). All analyses will be undertaken within the Secure Analytics Primary Health Environment, a controlled and audited secure computing environment, in accordance with Lumos data-use agreements and NSW Health information security requirements. Only de-identified, privacy-preserving linked data will be accessed, and no identifiable information will be extracted or exported.

This study involves secondary analysis of routinely collected health data and does not require direct participant consent. The study is conducted in accordance with established principles for prognostic model research and the secondary use of electronic health record data. The analytic strategy is prespecified to minimise selective reporting and reduce risks of data-driven model optimisation.

Results arising from this protocol will be reported in accordance with TRIPOD-AI guidance for prediction model development and validation, alongside relevant best-practice frameworks for reporting studies using routinely collected health data. Findings will be disseminated through peer-reviewed publications, presentations at national and international conferences and, where appropriate, plain-language summaries for clinical and community audiences. Any future clinical implementation would require additional co-design, software integration, safety assessment and prospective evaluation, which are outside the scope of this protocol.

No individual participants will be identifiable in any outputs.

## Data Availability

No data are available.
