# Supplementary material for: Dynamic prediction of cardiovascular risk from linked primary care records in New South Wales, Australia: protocol for a retrospective prognostic modelling study
Source: BMJ Open. 2026 Jul 27;16(7):e122980. doi: 10.1136/bmjopen-2026-122980 (PMC13409084; doi:10.1136/bmjopen-2026-122980)
Supplement: Supplementary file 1 [file bmjopen-2026-122980.reviewer_comments.pdf]

PEER REVIEW HISTORY

BMJ Open publishes all reviews undertaken for accepted manuscripts. Reviewers are asked to complete a checklist review form and are provided with free text boxes to elaborate on their assessment. These free text comments are reproduced below.

ARTICLE DETAILS

Title (Provisional)

Dynamic prediction of cardiovascular risk from linked primary care records in New South Wales, Australia: protocol for a retrospective prognostic modelling study

Authors

Kuo, Nicholas I-Hsien; Welberry, Heidi; Lujic, Sanja; Campain, Anna; Wilson, Daniel; Harris, Katie; Kidd, Michael; Correll, Patricia K; Peiris, David; Jorm, Louisa

---

VERSION 1 - REVIEW

**Reviewer**	**1**
**Name**	**Von Huben, Amy**
**Affiliation**	**The University of Sydney, Westmead Applied Research Centre**
**Date**	**16-Jun-2026**
**COI**	**None**

Describing "Existing CVD prediction tools are based on single static baseline assessments" seems not an altogether accurate description because these models are designed to be updated to the latest observations at the GP encounter with the patient. And the dynamic modelling approaches are only as dynamic as the recorded history and updating of the patient record allows. For example, it would be valuable to quantify the marginal benefit of a dynamic landmark prediction model updated with the Lumos data versus updating the current CVD risk tools like PREDICT with the patient; i.e., what is the marginal benefit of one model over the other based on how the models are envisaged to work in practice.

It would also be good to explain and examine how much of the expected marginal benefit from dynamic models is from just including time-varying predictors in the model (e.g., blood test results) and updating to the patient's most current observation versus using the individual's trajectory of these time-varying predictors over time in the model. At the moment these

two effects are combined together with no clear way to determine the benefit of each component.

The biggest concern for the study is around the data quality of the Lumos data, mainly in terms of completeness, to be used in a transformer-based sequence model. Interpreting the missingness as lack of clinical activity when there are so many other reasons why the data might be missing is a problem. A suggested approach for distinguishing between missing data because a patient has gone to another practice or the results have gone to another practice, versus actual patient clinical activity would be valuable to describe to gain some comfort around this issue.

Even with the dynamic landmark models, not having accurate smoking records, ethnicity and granular socioeconomic data is a risk for model misspecification. I think more needs to be written in the limitations for this - especially not having indicators for Aboriginal and Torres Strait Islander peoples. This model misspecification would only be able to be picked up with more out of time and out of sample testing (i.e., a different dataset than Lumos), which is not described in the protocol.

The results of Kuo et al 2025 give some comfort that the Lumos data may be able to provide a more predictive model than the current CVD risk tools available. Further work on these "non-dynamic" models to make them useable and practical for the GP and patient would be of great utility. There seems not enough explanation of in the protocol of how these new dynamic models would be implemented in practice and that is a risk in terms of them not being useable in practice.

The project would benefit greatly from an advisory group with a patient/consumer, GP, Cardiologist and GP software expert.

VERSION 1 - AUTHOR RESPONSE

Reviewer 1 comment 1

Comment: *Describing 'Existing CVD prediction tools are based on single static baseline assessments' seems not an altogether accurate description because these models are designed to be updated to the latest observations at the GP encounter with the patient. And the dynamic modelling approaches are only as dynamic as the recorded history and updating of the patient record allows. For example, it would be valuable to quantify the marginal benefit of a dynamic landmark prediction model updated with the Lumos data versus updating the current CVD risk tools like PREDICT with the patient; i.e., what is the marginal benefit of one model over the other based on how the models are envisaged to work in practice.*

Response: We agree that the previous wording could be misinterpreted as referring to how risk calculators are used in practice, rather than how most survival risk prediction models are developed. Our intended meaning was that these models are generally developed using a baseline survival modelling framework, in which each predictor is represented by a single value recorded at or near the start of follow-up. We have revised the abstract and introduction to clarify this distinction. We now explicitly acknowledge that tools such as PREDICT, QR4, PREVENT and AusCVDRisk can be re-run in clinical practice using the most recently available observations, while noting that the underlying models generally do not incorporate the longitudinal history of prior measurements, diagnoses, medication changes, consultation timing or gaps in recording. We have also clarified that dynamic models are limited by the completeness and updating of the recorded history, and we have strengthened the planned benchmark comparisons with existing risk algorithms and previously developed Lumos static risk models applied using the latest available encounter-level information where feasible.

Manuscript changes: Revised the abstract, introduction, aim, model development and model performance assessment sections to frame the key question as the marginal value of dynamic approaches over benchmark models updated with current encounter-level information.

Reviewer 1 comment 2

Comment: *It would also be good to explain and examine how much of the expected marginal benefit from dynamic models is from just including time-varying predictors in the model (e.g., blood test results) and updating to the patient's most current observation versus using the individual's trajectory of these time-varying predictors over time in the model. At the moment these two effects are combined together with no clear way to determine the benefit of each component.*

Response: We agree. We have revised the introduction and model development sections to distinguish between the potential benefit of updating predictors to current values and the additional potential benefit of modelling longitudinal trajectories and encounter patterns. The revised comparative analyses specify a staged comparison of: existing or previously developed static models applied using current encounter-level information; landmark models using time-updated covariates; landmark models including summaries of recent change or trajectory where feasible; and transformer-based sequence models using the ordered history.

Manuscript changes: Added text to the introduction, aim and model development sections specifying that the analyses will examine whether performance gains arise mainly from updating to current observations, including repeated quantitative clinical measurements, or modelling trajectories and encounter patterns.

Reviewer 1 comment 3

Comment: *The biggest concern for the study is around the data quality of the Lumos data, mainly in terms of completeness, to be used in a transformer-based sequence model. Interpreting the missingness as lack of clinical activity when there are so many other reasons why the data might be missing is a problem. A suggested approach for distinguishing between missing data because a patient has gone to another practice or the results have gone to another practice, versus actual patient clinical activity would be valuable to describe to gain some comfort around this issue.*

Response: We agree and have revised the missing data section to avoid implying that missingness equates to lack of clinical activity. The revised text states that missingness may

reflect selective recording, irregular encounter timing and care at practices that do not contribute data to Lumos. We now explicitly state that missingness will not be assumed to represent absence of clinical activity. We have added planned descriptive and sensitivity analyses using continuity-of-observation indicators, including time since first and last contributing-practice encounter, encounter frequency, long gaps between encounters, practice participation period and availability of repeated pathology or anthropometric measurements. For transformer models, the revised text states that intervals between recorded encounters may reflect a mixture of true clinical inactivity, care outside contributing practices and incomplete data capture.

Manuscript changes: Revised the 'Missing data and preprocessing' section to clarify interpretation of missingness and to add analyses intended to distinguish, as far as possible in routinely collected data, likely inactivity within contributing practices from incomplete capture due to care elsewhere.

Reviewer 1 comment 4

Comment: *Even with the dynamic landmark models, not having accurate smoking records, ethnicity and granular socioeconomic data is a risk for model misspecification. I think more needs to be written in the limitations for this - especially not having indicators for Aboriginal and Torres Strait Islander peoples. This model misspecification would only be able to be picked up with more out of time and out of sample testing (i.e., a different dataset than Lumos), which is not described in the protocol.*

Response: We agree. We have strengthened the manuscript to acknowledge that Aboriginal and Torres Strait Islander status, ethnicity, language and individual-level socioeconomic status are not available in Lumos, and that other variables such as smoking may be incompletely recorded. We have clarified that these limitations may restrict equity assessment and may obscure model misspecification in important population groups. We have also revised the internal-external validation section to state that leave-one-PHN-out validation should be interpreted as an assessment of transportability within Lumos rather than as a substitute for fully external validation in an independent data asset.

Manuscript changes: Revised the predictors, internal-external validation and subgroup analyses sections to state these limitations explicitly and to avoid overstating internal-external validation as independent external validation.

Reviewer 1 comment 5

Comment: *The results of Kuo et al 2025 give some comfort that the Lumos data may be able to provide a more predictive model than the current CVD risk tools available. Further work on these 'non-dynamic' models to make them useable and practical for the GP and patient would be of great utility. There seems not enough explanation of in the protocol of how these new dynamic models would be implemented in practice and that is a risk in terms of them not being useable in practice.*

Response: We agree that implementation feasibility is central. We have clarified that the current study will produce validated risk prediction models and evidence on comparative performance, calibration, clinical utility and transportability, but will not implement a live tool in GP software. We have added an 'Implementation considerations' subsection addressing predictor availability in GP records at the prediction encounter, computational requirements for encounter-level predictions, explainability of model outputs for clinicians

and patients, workflow burden and the trade-off between incremental performance and implementation complexity. We have also added text in the Ethics and dissemination section clarifying that future clinical implementation would require additional co-design, software integration, safety assessment and prospective evaluation, which are outside the scope of this protocol.

Manuscript changes: Added an 'Implementation considerations' subsection and revised related text in the introduction, model development, model performance assessment, patient and public involvement, and ethics and dissemination sections.

Reviewer 1 comment 6

Comment: *The project would benefit greatly from an advisory group with a patient/consumer, GP, Cardiologist and GP software expert.*

Response: We agree that advisory input is important. The broader Lumos program and this research project are informed by established governance and advisory structures, including a Steering Committee with consumer representation, GP expertise and GP software expertise, and a dedicated Consumer Reference Group. We have revised the Patient and public involvement section to describe these structures and their role in prioritisation, oversight of data use, interpretation and communication of findings, and consideration of community expectations regarding privacy, data sharing and public benefit.

Manuscript changes: Revised the Patient and public involvement section to describe the Steering Committee and Consumer Reference Group, including consumer, GP and GP software perspectives.

We hope these revisions address the editor and reviewer comments.

Sincerely,

Louisa Jorm, on behalf of all authors
